# Effects of Pirimiphos-Methyl on Non-Target Invertebrates

**DOI:** 10.3390/biology13100823

**Published:** 2024-10-14

**Authors:** Liudmyla Faly, Viktor Brygadyrenko

**Affiliations:** 1 Research Institute of Natural and Technological Sciences, Department of Biology, Faculty of Natural Sciences, Vytautas Magnus University, K. Donelaičio Str. 58, 44248 Kaunas, Lithuania; liudmyla.faly@vdu.lt; 2 Department of Zoology and Ecology, Faculty of Biology and Ecology, Oles Honchar Dnipro National University, Gagarin Av. 72, 49010 Dnipro, Ukraine; 3 Department of Parasitology, Veterinary and Sanitary Expertise, Faculty of Veterinary Medicine, Dnipro State Agrarian and Economic University, Sergiy Efremov St. 25, 49600 Dnipro, Ukraine

**Keywords:** non-target invertebrates, phosphorus-organic insecticides, pirimiphos-methyl, sensitivity, tolerance to insecticide, semi-lethal dose, survival of species

## Abstract

**Simple Summary:**

The widespread use of broad-spectrum insecticides reduces the capabilities of ecological self-regulation of phytophages in agrocoenoses and in the adjacent natural territories. Those ecosystems suffer disruption of trophic relations that have been forming over long periods of time. At the same time, the numbers of non-target invertebrates affected by pesticides are declining. Pirimiphos-methyl is one of the cheapest, most effective, and widely used insecticides in the world. Its effect on invertebrate species is useful for agriculture; however, its effect on arthropods that improve soil fertility remains virtually unstudied. This article establishes that this insecticide can have a strong effect on some species useful for plant growing and a weak effect on others. As a result of such an effect of pirimiphos-methyl, the taxonomic structure of invertebrate communities in agrocenoses should change dramatically. The use of this insecticide in fields gives an advantage to species that are relatively more resistant to it and causes a faster disappearance of sensitive invertebrate species.

**Abstract:**

The effects of pirimiphos-methyl have previously been assessed on blood-sucking insect species, pollinating insects, and target crop pest species. The sensitivity of non-target zoophagous and saprophage species to this insecticide remains largely unstudied. In laboratory conditions, we assessed the susceptibility of 43 species of invertebrates to pirimiphos-methyl. The most tolerant species to this insecticide were *Pyrrhocoris apterus* (LC_50_ measured over 60 mg/m^2^), *Cylindroiulus truncorum*, *Pterostichus niger*, *Harpalus rufipes*, *Lithobius forficatus*, and *Carabus hortensis* (LC_50_ ranged from 25 to 50 mg/m^2^). Average tolerance to pirimiphos-methyl was displayed by *Ophonus rufibarbis*, *Teuchestes fossor*, *Silpha carinata*, *Badister sodalis*, *Rugilus rufipes*, *Phosphuga atrata*, *Porcellio laevis*, *Pterostichus oblongopunctatus*, *Aphodius foetens*, *Lasius fuliginosus*, *Oxypselaphus obscurus*, *Platydracus fulvipes*, *Myrmica ruginodis*, *Xantholinus tricolor*, and *Megaphyllum* sp. (LC_50_ for those species ranged from 12 to 24 mg/m^2^). Higher sensitivity to this insecticide was seen for *Amara nitida*, *Leistus ferrugineus*, *Harpalus xanthopus winkleri*, *Philonthus nitidus*, *Pterostichus melanarius*, *Harpalus latus*, *Limodromus assimilis*, *Philonthus decorus*, *Tachinus signatus*, *Ponera coarctata*, *Carabus convexus*, *Philonthus coprophilus*, *Philonthus laevicollis*, *Platydracus latebricola*, *Labia minor*, and *Carabus granulatus* (LC_50_ for those species ranged from 6 to 12 mg/m^2^). The greatest sensitivity to pirimiphos-methyl was observed in *Hister fenestus*, *Drusilla canaliculata*, *Bisnius fimetarius*, *Oxytelus sculptus*, *Lasius niger*, and *Lasius flavus* (LC_50_ ranged from 0.4 to 6 mg/m^2^). We found a relationship between the parameters of bodies of invertebrates (the average body length and dry body mass) and sensitivity to pirimiphos-methyl. With an increase in body sizes of invertebrates, the tolerance to the insecticide increased (per each mm of body length, LC_50_ increased by 0.82 mg/m^2^ on average). We identified no relationship between the trophic specialization and sensitivity to the insecticide.

## 1. Introduction

The problem of preservation of biological diversity becomes increasingly relevant each year. Insecticides are an important tool against invertebrate pests. Chemical means of protection of plants destroy harmful pests, but malpractice disrupts the ecological balance, impoverishing the beneficial entomofauna [1,2]. Therefore, the effects of insecticides on the populations of non-target invertebrates in agrocoenoses and forest ecosystems require in-detail analysis [3,4]. As of now, various influence mechanisms of chemical means of plant protection have been described. However, their main action comes down to disruption of important biological processes in invertebrates. Various classes of preparations with insecticide activity are considerably different, working by selective action, i.e., they have selective toxicity. Having high selective action, the active agent only affects a particular pest, not harming other species, even if they are in close contact, or selectively impacts particular metabolic processes. The action of modern pesticides is oriented at specific proteins—targets that participate in certain physiological processes. Those proteins are unique in each group of harmful invertebrates [5]. Selectivity of a toxicant is determined by the method by which the active compound is used, morphological and behavioral peculiarities of organisms, specifics of penetration, transformation, and release of the pesticide [6]. Ecological-toxic principles of the development of the chemical method of plant protection are aimed at the search for and use of chemical drugs with selective action which minimizes the effects on non-target species [7,8].

The Insecticide Resistance Action Committee (IRAC) identifies four main categories of chemicals (which are further divided into numerous subgroups) by their action mechanism [9]. The most common category on the global market is preparations with broad-spectrum activity—insecticides that paralyze the nervous-muscular processes. Then, there are insecticides that affect the growth and development of insects, insecticides of respiratory action, and means of plant protection that irritate the intestinal tract of insects [10]. Considering the large variety of preparations with insecticidal action, reactions of some taxonomic groups of invertebrates to insecticides with different action mechanisms differ greatly [11,12].

In communities of agrocoenose invertebrates, the target pests are typically one, and rarely two to five, species. Hundreds of other invertebrates living in one hectare of field, garden, or forest suffer from insecticides to a greater or lesser degree [13].

An acute issue of nature protection at the global level is protection of globally endangered invertebrates (https://iucnredlist.org, accessed on 30 January 2024). Usually, when describing the causes of why a beetle or hymenopteran is vanishing, the authors tend to only indicate neutral phrases (“vanishing of living locations”, “pollution of environment with pesticides”, “pesticides from adjacent agrocoenoses impacting the ecosystems where populations of this species live”, etc.) [14].

One of the most globally used insecticides is pirimiphos-methyl (O-2-diethylamino-6-methylpyrimidin-4-yl O,O-dimethyl phosphorothioate). It belongs to the group of organophosphorous compounds with insecticide and acaricide activities.

It is a broad-spectrum insecticide with contact and respiratory action, and also an acetylcholinesterase (AChE) inhibitor [15]. Once the activity of this enzyme is suppressed, acetylcholine accumulates in the synaptic cleft and the work of ion channels of the post-synaptic membrane becomes prolonged, ultimately causing paralysis and death of the invertebrate [16]. Pirimiphos-methyl was developed by the British chemical company Imperial Chemical Industries (ICI) in the late 1960s and debuted on the market in 1977. The most common drug in European countries that contains this compound is Actellic (500 g/L emulsion). It is used for the protection of vegetable and ornamental plants in open areas and in greenhouses from leaf-eating and sucking harmful insects and acarians, and also for sanitary treatment of warehouses and grain facilities from storage pests. The drug is effective against harmful arthropods that are resistant to pyrethroids and carbamates and is non-carcenogenic. Pirimiphos-methyl belongs to toxicity class II, being highly detrimental to aquatic organisms [17,18].

Many acarians and insects have developed a tolerance to this pesticide, for example, *Acarus chaetoxysilus*, *Acarus farris*, *Blattella germanica*, *Ephestia cautella*, *Meligethes aeneus*, and many others [19]. This compound is quite stable in the conditions of natural ecosystems: its concentration reduced by a factor of two in 18–67 days in field conditions, compared with 3–21 days in laboratory conditions [20]. Despite the broad application of this pesticide in many countries around the world, its effects on many insects have been reported fragmentarily: contact acute LD_50_ (worst case from 24, 48, and 72 h values)—0.27 μg/bee for *Apis mellifera*; oral acute LD_50_ (worst case from 24, 48, and 72 h values)—>0.22 μg/bee for *Apis mellifera* [21].

The objective of this study was laboratory assessment of the effects of pirimiphos-methyl on non-target invertebrates living in forests and meadow ecosystems of Europe.

## 2. Materials and Methods

The field material was collected in the forest and meadow ecosystems around the city of Kaunas (Lithuania) in the period from May to July 2024. The invertebrates were caught using the generally accepted methods: Barber pitfall traps, manually from the litter and soil, aspirators, and butterfly nets [22,23].

In the laboratory experiments, we used 43 species of invertebrates of various taxonomic groups. The experiments were conducted in the laboratory, in stationary conditions: a temperature of 21–23 °C and a relative air humidity of 45–55%. In order to measure the susceptibility to pirimiphos-methyl, the invertebrates were put into small containers (10 cm × 6 cm × 4 cm) with lids of thin food-grade plastic. Onto the bed of each container, we put several cotton swabs for absorption of excessive moisture. The number of invertebrates in a container varied depending on their body sizes: large ground beetles—4 individuals in a container, smaller species—8 specimens. In the experiment, only mature specimens of invertebrates participated. Using household plastic pulverizers, into each container we introduced the same dose of pirimiphos-methyl (the drug Actellic 500 EC, Basel, Switzerland)—0.37 mL of aqueous solution in certain concentrations. We used 11 concentrations of the insecticide (800, 200, 100, 50, 25, 12.5, 6.25, 4.167, 3.125, 1.563, 0.781 mg/m^2^). For each concentration, 5–8 replicates were used (Table 1). The microscopic drops of aqeous solution of pirimiphos-methyl touched, with the same likelihood, the coatings of the arthropods, cotton swabs on the bed, and the bed and walls of the plastic container. According to the manual, the manufacturer-recommended dose of pirimiphos-methyl in agrocoenoses is 50 mg/m^2^ or 500 g/ha (Table 2). The containers filled with the arthropods were placed in laboratory fume exhaust hood for 24 h. Then, the live and dead specimens were counted (Table 1). Individuals with no signs of life or individuals with obvious visible disturbances in the functioning of the nervous system (limited mobility, unnatural body position, tremors of the limbs and antennae) were considered dead. According to our observations, invertebrates with signs of toxic damage died in the following few days after the experiment. Dead invertebrates were fixated in 70% ethanol and placed into the collection funds of the university.

We prepared the control groups of invertebrates for the experiment in the same way as the experimental groups. For the control groups, the same amount (0.37 mL) of distilled water was added to the containers with a spray instead of an aqueous solution of insecticide and left for 24 h. The survival rate of individuals in the control groups for all studied species was 100%.

The statistical analysis of the results was performed through a set of Statistica 12.0 (StatSoft Inc., Tulsa, OK, USA). The results were analyzed using probit analysis. The samplings were compared using the Tukey test prior to the analysis (ANOVA) with the Bonferroni correction. To assess the dependence of LC_50_ on the body length and body weight of invertebrates, we used regression analysis (the relationship between the dependent and independent variables was assessed using the coefficients of determination R^2^). Table 2 and the text provide the mean values (x) ± standard error (SE) for LC_50_. 

## 3. Results

According to results of our laboratory experiments (Table 1 and Table 2), LC_50_ within one family can vary by up to 10 times. The dose of pirimiphos-methyl recommended by the manufacturer is 50 mg/m^2^. The median lethal dose for *Pyrrhocoris apterus* exceeded this value, measuring 62.63 ± 10.34 mg/m^2^ (Table 2).

Relative tolerance to this insecticide (LC_50_ ranging 50% to 100% of the dose recommended by the manufacturer) was exerted by *Cylindroiulus truncorum* (LC_50_ was 49.22 ± 10.35 mg/m^2^), *Pterostichus niger* (30.29 ± 6.15), *Harpalus rufipes* (28.14 ± 5.68), *Lithobius forficatus* (27.70 ± 2.68), and *Carabus hortensis* (25.25 ± 3.76, Table 2).

The LC_50_ range of 25% to 50% of the dose recommended by the manufacturer was observed for *Ophonus rufibarbis* (22.37 ± 4.15 mg/m^2^), *Teuchestes fossor* (22.12 ± 4.21), *Silpha carinata* (21.91 ± 5.00), *Badister sodalis* (19.24 ± 3.69), *Rugilus rufipes* (19.01 ± 3.30), *Phosphuga atrata* (18.55 ± 3.22), *Porcellio laevis* (18.24 ± 3.08), *Pterostichus oblongopunctatus* (18.20 ± 2.80), *Aphodius foetens* (15.14 ± 3.18), *Lasius fuliginosus* (14.96 ± 2.53), *Oxypselaphus obscurus* (14.67 ± 2.81), *Platydracus fulvipes* (14.64 ± 3.32), *Myrmica ruginodis* (14.32 ± 2.58), *Xantholinus tricolor* (13.25 ± 1.48), and *Megaphyllum* sp. (12.95 ± 1.94). Those species had average resistance to this insecticide.

High sensitivity to pirimiphos-methyl (LC_50_ ranging 12.5% to 25.0% of the dose recommended by the manufacturer) was seen in species such as *Amara nitida* (12.03 ± 2.81 mg/m^2^), *Leistus ferrugineus* (10.56 ± 2.12), *Harpalus xanthopus winkleri* (10.27 ± 2.16), *Philonthus nitidus* (9.68 ± 2.66), *Pterostichus melanarius* (9.62 ± 1.97), *Harpalus latus* (9.56 ± 1.90), *Limodromus assimilis* (9.23 ± 2.58), *Philonthus decorus* (9.23 ± 2.58), *Tachinus signatus* (9.00 ± 1.44), *Ponera coarctata* (8.98 ± 2.42), *Carabus convexus* (8.74 ± 1.43), *Philonthus coprophilus* (8.58 ± 2.44), *Philonthus laevicollis* (8.14 ± 2.30), *Platydracus latebricola* (8.02 ± 1.92), *Labia minor* (7.91 ± 1.94), and *Carabus granulatus* (7.58 ± 1.46).

The highest sensitivity to this insecticide (LC_50_ below 12.5% of the dose recommended by the manufacturer) was observed in the following species: *Hister fenestus* (5.32 ± 1.14 mg/m^2^), *Drusilla canaliculata* (4.63 ± 1.19), *Bisnius fimetarius* (2.12 ± 0.70), *Oxytelus sculptus* (0.756 ± 0.328), *Lasius niger* (0.603 ± 0.234), and *Lasius flavus* (0.481 ± 0.171).

Thus, of 43 studied invertebrates, LC_50_ ranged over 100% of the dose recommended by the manufacturer for one species, ranged 50–100% for five species, 25–50% for 15 species, 12.5–25.0% for 16 species, 6.25–12.50% for two species, and 6.25% for four species (Table 2).

**Figure 1:**
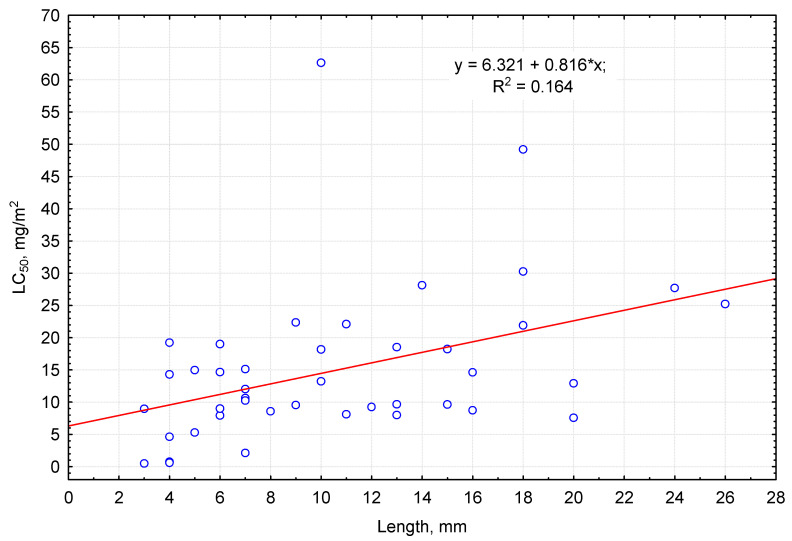
Relationship between LC_50_ of pirimiphos-methyl (ordinate axis) and the average body length of the invertebrates (abscissa axis).

The median lethal dose for pirimiphos-methyl increased with an increase in invertebrates’ body length (Figure 1). For 2–4-mm-long invertebrates, LC_50_ ranged up to 19 mg/m^2^. With increase in body length by 1 mm, LC_50_ on average increased by 0.82 mg/m^2^ (see equation of linear regression in Figure 1). Invertebrates with 4–6 mm body length had average tolerance to pirimiphos-methyl. The highest tolerance to this insecticide in this size group was observed in small ground beetles (*Badister sodalis* and *Oxypselaphus obscurus*—LC_50_ measured up to 20 mg/m^2^). Among species of the average size group, with the body length up 13 mm, the highest tolerance was seen in hemipterans (*Pyrrhocoris apterus* LC_50_ of over 60 mg/m^2^) and coleopterans (*Ophonus rufibarbis*, *Teuchestes fossor*, *Pterostichus oblongopunctatus*, *Rugilus rufipes,* and *Bisnius fimetarius).* The maximum sensitivity was recorded for *Bisnius fimetarius* (LC_50_ of up to 3 mg/m^2^). Large arthropods with up to 30 mm body length were characterized as averagely or highly tolerant to pirimiphos-methyl, for example, millipedes *Lithobius forficatus* and *Cylindroiulus truncorum:* the median lethal doses for them accounted for 27.7 and 49.2 mg/m^2^, respectively. The same category included large ground beetles, staphylinids, and silphids (*Pterostichus niger*, *Harpalus rufipes*, *Carabus hortensis*, *Platydracus fulvipes*, *Silpha carinata*, the LC_50_ equaling 14–31 mg/m^2^).

**Figure 2:**
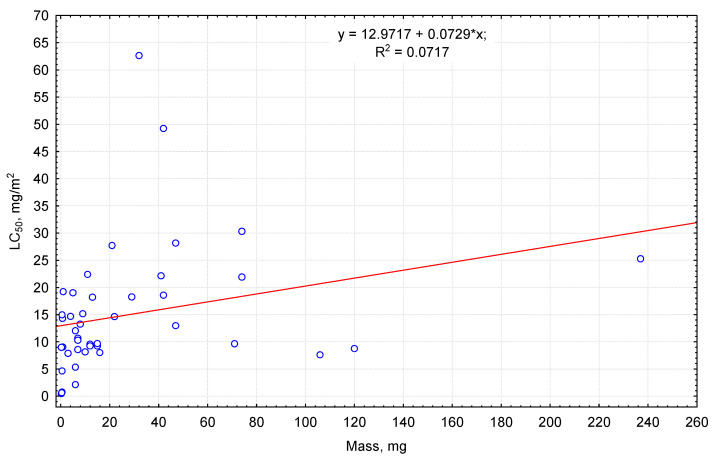
Relationship of LC_50_ to pirimiphos-methyl (ordinate axis) and mean dry mass of the invertebrates (abscissa axis).

A similar, but less expressed, tendency was seen for body mass of the invertebrates: individuals with higher body mass had higher LC_50_ values (Figure 2). The median lethal dose for arthropods whose dry weight was above 20 mg was on average 24 mg/m^2^. Exceptions were large groundbeetles of the *Carabus* genus with over 100 mg body mass (*Carabus convexus*, *C. granulatus*—their LC_50_ equaled 7–9 mg/m^2^). Arthropods with 1–16 mg dry body mass can be characterized as moderately sensitive, the median lethal doses for them equaling around 10 mg/m^2^. Species with below 1 mg body mass were represented in our study by small staphylinids and ants. The same weight group, according to results of the experiments, included both averagely sensitive and the most sensitive to pirimiphos-methyl (ants of the *Lasius* genus with an LC_50_ of 0.4–0.6 mg/m^2^).

**Figure 3:**
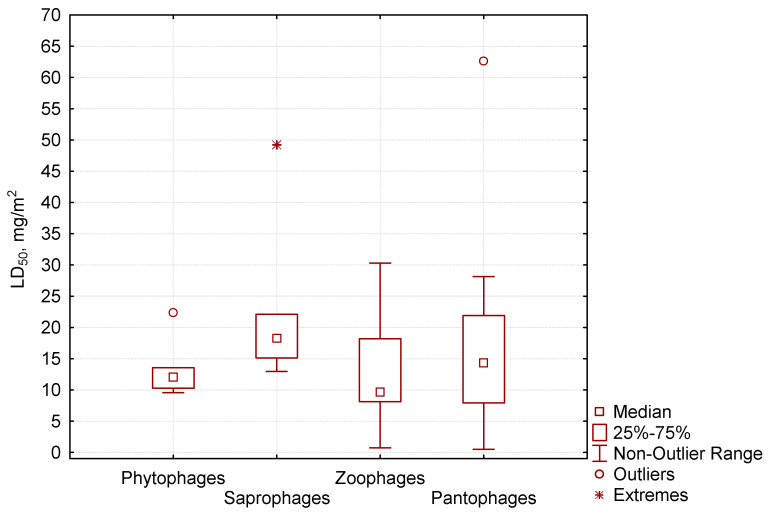
Relationship between LC_50_ to pirimiphos-methyl (ordinate axis) for various trophic groups.

Of the four trophic groups of invertebrates that we studied (Figure 3), the median of the LC_50_ value for phytophages was 12.6 mg/m^2^, 18.2 mg/m^2^ for saprophages, 9.7 mg/m^2^ for zoophages, and 14.3 mg/m^2^ for pantophages. Saprophages and pantophages included invertebrates with the highest identified resistance to pirimiphos-methyl. Saprophages were represented by the Porcellionidae woodlice, the Julidae millipedes, and coleopterans by the Aphodiidae coprophages, which were quite resistant to the insecticide. The group of pantophages was formed of the Spongiphoridae earwigs, the Pyrrhocoridae hemipterans, and some species of the Carabidae and Silphidae coleopterans, and the Formicidae ants. Those groups are very diverse taxonomically. The sensitivity to insecticide in various groups varied significantly, and therefore we saw drastically different values of the median lethal dose. Among the mentioned invertebrates, some species were characterized by maximum tolerance to pirimiphos-methyl, while others had maximum sensitivity.

In our study, phytophages were not numerous. This group included some species of Carabidae that were moderately sensitive to the insecticide. The zoophages had the lowest values of median lethal dose (the median below 10 mg/m^2^). This group mostly comprised predatory coleopterans, including species of Staphylinidae that had the highest sensitivity to pirimiphos-methyl. In our similar studies with cypermethrin, we observed a similar reaction: staphylinids exhibited minimal tolerance to this pyrethroid. In general, we found no clear correlation between the value of median lethal dose of pirimiphos-methyl and the classification of an invertebrate into a specific trophic group.

Three families of invertebrates in our experiment were represented by 5–15 species; therefore, it was possible to analyze the reliability of differences in half-lethal concentrations of the insecticide for these groups. The LC_50_ for 15 species of the Carabidae family was maximum (15.7 ± 2.0 mg/m^2^). The LC_50_ value (7.9 ± 3.2 mg/m^2^) for 5 species of the Formicidae family was insignificantly lower (*p*-value = 0.059, F = 4.069, F_0.05_ = 4.414). The LC_50_ value for 12 species of the Staphylinidae family (8.9 ± 1.5 mg/m^2^) was significantly different from the Carabidae family (*p*-value = 0.014, F = 6.955, F_0.05_ = 4.242).

## 4. Discussion

Pirimiphos-methyl is one of the most effective, most popular, and cheapest plant protection products. At the same time, differences in LC_50_ for various species ranged significantly less than in case of the pyrethroid that we studied earlier, cypermethrin [24], subject to which variations in LC_50_ for different invertebrates exceeded 100,000-fold. The minimal sensitivity to both cypermethrin and pirimiphos-methyl in our experiments was exhibited by *Pterostichus niger*, while the highest sensitivity to cypermethrin among all the studied coleopterans was displayed by staphylinids (*Philonthus decorus*, *Tachinus signatus*, *Oxytelus sculptus*). The same species were the most sensitive to pirimiphos-methyl.

Similar results were recorded for some common species of ants (*Lasius niger*). However, our studies suggest that drawing comparisons of the effects of insecticides from various chemical classes on the same invertebrates may reveal significant differences in their tolerance. For example, the firebugs *Pyrrhocoris apterus* had the maximum tolerance to pirimiphos-methyl. The median lethal dose for this species exceeded the dose recommended for usage in agrosystems. However, in the experiments with cypermethrin, this species exerted high sensitivity. The similar situation was seen for the millipedes *Cylindroiulus truncorum* and the groundbeetles *Carabus hortensis*, which turned out to have the maximum tolerance to organophosphorus insecticides on the example of pirimiphos-methyl and were quite sensitive to pyrethroids (cypermethrin).

Species such as *Philonthus rectangulus*, *Ph. decorus*, *Oxytelus sculptus*, *Ophonus rufibarbis*, *Carabus granulatus*, *C. hortensis*, *Myrmica ruginodis*, and some others were killed by cypermethrin at concentrations of 100–10,000 times lower than those recommended for agrocoenoses [24]. At the same time, *Lasius flavus*, *L. niger*, *Bisnius fimetarius*, *Drusilla canaliculata*, *Hister fenestus*, and some others died subject to 10–50 times lower concentrations than those recommended for field usage by manufacturers of pirimiphos-methyl.

Obviously, insecticides and other synthetic pollutants can transform a community of invertebrates [3,24]. Species that are sensitive to a particular insecticide will be the first to disappear in such communities, for example, many staphylinids, ants, some groundbeetles, and hemipterans. More tolerant species, such as *Pterostichus niger*, *P. melanarius*, *Calathus ambiguous*, and some others, receive a competitive advantage on the field peripheries and gardens, where the concentration of insecticide is not sufficient to kill those species, while their competitors become unable to effectively sustain their numbers.

Populations of *Sitophilus oryzae* [25,26], *Oryzaephilus surinamensis* [27], *Tribolium castaneum* [28], *Rhyzopertha dominica* [29], *Typhaea stercorea* [30], *Musca domestica* [31,32], *Lucilia sericata* [33], *Tetranychus urticae* [34], and other species can adapt to pirimiphos-methyl due to the formation of altered conformations of enzymes, while the active sites of these molecules cease to effectively interact with the pesticide. The natural variability in insect populations results in each generation containing a certain percentage (denoted further as x) of individuals that can survive exposure to pirimiphos-methyl. If the number of generations per year for this species is considerably high, and pirimiphos-methyl continuously affects the populations over several years, then x% of specimens will survive in the first generation. Given that various invertebrates produce an average of 20–200 offspring (approximately 100 successors) in the next generation, two generations later (with continued use of this insecticide), the population will consist of 10,000 × x% stable genotypes; three generations later, it will comprise 1,000,000 × x%; four generations later, it will reach 100,000,000 × x%; and so on. Regardless of how low the value of x (% of non-sensitive specimens in the insect generation) is, after 5–10 generations, the population will be predominantly composed of such forms.

At the same time, rare species of invertebrates typically have long development periods from egg to oviposition (for example, *Lucanus cervus* and *Cerambyx cerdo* develop over 5 to 7 years). Therefore, the spread of tolerance to certain pesticides within their populations is very slow compared with pests such as *Leptinotarsa decemlineata*, which can produce three generations a year. Furthermore, study of the spread of insecticide resistance in non-target invertebrates is an extremely laborious process, to which a low number of reports has been dedicated [3].

Pirimiphos-methyl is broadly used against blood-sucking mosquitoes *Aedes aegypti* [35], *Anopheles gambiae* [36,37], mosquitoes *Phlebotomus argentipes* [38], bed bugs *Cimex lectularius* [39], house flies [40,41], cabbage aphids *Brevicoryne brassicae* [42], and also storage pests such as *Trogoderma granarium* [43,44], *Tenebrio molitor* [45,46], *Callosobruchus chinensis*, *Sitophilus zeamais* [47], *Cryptolestes ferrugineus* [48], and *Ephestia kuehniella* [49]. Non-target species that are resistant to this insecticide have existed since the late 1970s in many regions of the world, due to large-scale usage of pesticides, including dispersal from aircraft, like for example, against *Anopheles* [50]. We collected non-target species of invertebrates on plots located at a great distance from agrocoenoses—in forests and meadow ecosystems of Kaunas District of Lithuania, which, according to the data of nature-protection bodies and locals, have never been subjected to pesticide treatments. Therefore, we expect that our results reflect the natural sensitivity of the species to pirimiphos-methyl, which had not been shaped by previous exposure to pesticides.

Potential limitations of this study should also be discussed. Since our experiments were conducted at a constant temperature of 21–23 °C, which differs from natural conditions in agrocenoses, the LC_50_ values under field and garden conditions for each species may be higher than at temperatures of 10–13 °C or lower than at temperatures of 30–33 °C. With an increase in environmental temperature, the intensity of the effect of an insecticide or other biologically active substance increases. In nature, invertebrates can migrate freely (both vertical movements between the grass stand, litter, and soil, and horizontal migrations to areas bordering the agrocenosis) in the presence of unfavorable anthropogenic influences (e.g., treatment of fields with insecticides). This also limits the application of the results of laboratory experiments. The rate of microbiological degradation of the insecticide by soil bacteria and fungi depends on abiotic environmental factors and will differ significantly between laboratory and natural conditions. Heavy precipitation also reduces the concentration of the insecticide and promotes its leaching from the soil. Thus, our laboratory experiments assess the standard tolerance of each arthropod species to the insecticide under strictly controlled laboratory conditions. Under the influence of spatial and temporal heterogeneity of temperature and humidity in agrocenoses, the resistance of individual species in agrocenoses and laboratory conditions may differ.

## 5. Conclusions

In laboratory conditions, we assessed the susceptibility of 43 species of invertebrates of various taxonomic groups to the broadly used agricultural organophosphate insecticide pirimiphos-methyl. The studies revealed no distinct interrelation between the trophic specialization and sensitivity to the insecticide, and therefore we should consider individual species sensitivity. We found maximum tolerance to pirimiphos-methyl in the firebug *Pyrrhocoris apterus* (LC_50_ measuring over 60 mg/m^2^). High tolerance was characteristic of the millipedes *Cylindroiulus truncorum* and *Lithobius forficatus* and the groundbeetles *Pterostichus niger*, *Harpalus rufipes* and *Carabus hortensis*, the median lethal dose for which ranged 25–50 mg/m^2^. Among moderately sensitive species, for which LC_50_ accounted for 12 to 25 mg/m^2^, we should note *Ophonus rufibarbis*, *Teuchestes fossor*, *Silpha carinata*, *Badister sodalis*, *Rugilus rufipes*, *Phosphuga atrata*, *Porcellio laevis*, *Pterostichus oblongopunctatus*, *Aphodius foetens*, *Lasius fuliginosus*, *Oxypselaphus obscurus*, *Platydracus fulvipes*, *Myrmica ruginodis*, *Xantholinus tricolor*, *and Megaphyllum* sp. Greater sensitivity to pirimiphos-methyl was seen in *Amara nitida*, *Leistus ferrugineus*, *Harpalus xanthopus winkleri*, *Philonthus nitidus*, *Pterostichus melanarius*, *Harpalus latus*, *Limodromus assimilis*, *Philonthus decorus*, *Tachinus signatus*, *Ponera coarctata*, *Carabus convexus*, *Philonthus coprophilus*, *Ph. laevicollis*, *Platydracus latebricola*, *Labia minor*, and *Carabus granulatus* (LC_50_ measuring 6–12 mg/m^2^). The highest sensitivity to the insecticide was observed for *Hister fenestus*, *Drusilla canaliculata*, *Bisnius fimetarius*, *Oxytelus sculptus*, *Lasius nige*, and *L. flavus* (LC_50_ 0.4–6.0 mg/m^2^).

The interrelation between body length of invertebrates and sensitivity to pirimiphos-methyl was less expressed. However, we observed a tendency for larger body mass in invertebrates to be associated with a higher LC_50_. For large arthropods with a body mass of over 20 mg, LC_50_ was on average 24 mg/m^2^. For the smaller weight category, 1–16 mg, LC_50_ was on average around 10 mg/m^2^. In the group of invertebrates with up to 1 mg body mass, we found the most sensitive species to pirimiphos-methyl, for which LC_50_ was no higher than 0.6 mg/m^2^.

The results of our experiments demonstrated that invertebrates that belong to different taxonomic groups significantly vary in sensitivity to pirimiphos-methyl. Application of this pesticide in agrocoenoses and forests leads to considerable transformation of local communities of invertebrates and changes in competitive interactions between species with similar trophic preferences: in territories contaminated with this insectoacaricide, tolerant species acquire advantages, and the taxonomic composition of the communities of invertebrates are significantly impoverished.

## Figures and Tables

**Table 1:** Results of laboratory experiments with 24 h exposure of the invertebrates to pirimiphos-methyl.

Order	Family	Species	Result of the Experiment	800 mg/m^2^	200 mg/m^2^	100 mg/m^2^	50 mg/m^2^	25 mg/m^2^	12.5 mg/m^2^	6.25 mg/m^2^	4.167 mg/m^2^	3.125 mg/m^2^	1.563 mg/m^2^	0.781 mg/m^2^
Isopoda	Porcellionidae	*Porcellio laevis* Latreille, 1804	Living	0	0	0	0	3	7	6	8	7	8	8
			Dead	8	8	8	8	5	1	2	0	1	0	0
Lithobiomorpha	Lithobiidae	*Lithobius forficatus* (Linnaeus, 1758)	Living	0	0	1	3	5	6	7	8	7	8	8
			Dead	8	8	7	5	3	2	1	0	1	0	0
Julida	Julidae	*Cylindroiulus truncorum* (Silvestri, 1896)	Living	0	0	2	4	7	8	8	8	8	8	8
			Dead	8	8	6	4	1	0	0	0	0	0	0
		*Megaphyllum* sp.	Living	0	0	0	1	1	2	4	7	8	8	8
			Dead	8	8	8	7	7	6	4	1	0	0	0
Dermaptera	Spongiphoridae	*Labia minor* (Linnaeus, 1758)	Living	0	0	0	0	0	0	3	5	5	6	6
			Dead	6	6	6	6	6	6	3	1	1	0	0
Hemiptera	Pyrrhocoridae	*Pyrrhocoris apterus* (Linnaeus, 1758)	Living	0	2	3	4	5	8	8	8	8	8	8
			Dead	8	6	5	4	3	0	0	0	0	0	0
Coleoptera	Carabidae	*Carabus convexus* Fabricius, 1775	Living	0	0	0	0	1	1	3	6	7	8	8
			Dead	8	8	8	8	7	7	5	2	1	0	0
		*C. granulatus* Linnaeus, 1758	Living	0	0	0	0	0	2	2	5	7	8	8
			Dead	8	8	8	8	8	6	6	3	1	0	0
		*C. hortensis* Linnaeus, 1758	Living	0	0	0	1	2	4	5	5	5	5	5
			Dead	5	5	5	4	3	1	0	0	0	0	0
		*Leistus ferrugineus* (Linnaeus, 1758)	Living	0	0	0	0	0	2	2	4	5	5	5
			Dead	5	5	5	5	5	3	3	1	0	0	0
		*Pterostichus niger* (Schaller, 1783)	Living	0	0	0	1	3	5	5	5	5	5	5
			Dead	5	5	5	4	2	0	0	0	0	0	0
		*P. melanarius* (Illiger, 1798)	Living	0	0	0	0	0	2	3	6	8	8	8
			Dead	8	8	8	8	8	6	5	2	0	0	0
		*P. oblongopunctatus* (Fabricius, 1787)	Living	0	0	0	0	2	5	7	8	8	8	8
			Dead	8	8	8	8	6	3	1	0	0	0	0
		*Limodromus assimilis* (Paykull, 1790)	Living	0	0	0	0	0	0	4	7	8	8	8
			Dead	8	8	8	8	8	8	4	1	0	0	0
		*Ophonus rufibarbis* (Fabricius, 1792)	Living	0	0	0	0	2	4	5	5	5	5	5
			Dead	5	5	5	5	3	1	0	0	0	0	0
		*Badister sodalis* (Duftschmid, 1812)	Living	0	0	0	0	1	4	6	6	6	6	6
			Dead	6	6	6	6	5	2	0	0	0	0	0
		*Harpalus latus* (Linnaeus, 1758)	Living	0	0	0	0	0	2	4	5	8	8	8
			Dead	8	8	8	8	8	6	4	3	0	0	0
		*H. rufipes* (DeGeer, 1774)	Living	0	0	0	1	2	5	5	5	5	5	5
			Dead	5	5	5	4	3	0	0	0	0	0	0
		*H. xanthopus winkleri* Schauberger, 1923	Living	0	0	0	1	0	1	4	6	8	8	8
			Dead	8	8	8	7	8	7	4	2	0	0	0
		*Amara nitida* Sturm, 1825	Living	0	0	0	0	0	1	3	5	5	5	5
			Dead	5	5	5	5	5	4	2	0	0	0	0
		*Oxypselaphus obscurus* (Herbst, 1784)	Living	0	0	0	0	1	1	4	5	5	5	5
			Dead	5	5	5	5	4	4	1	0	0	0	0
Coleoptera	Staphylinidae	*Tachinus signatus* Gravenhorst, 1802	Living	0	0	0	0	0	2	3	3	4	5	5
			Dead	5	5	5	5	5	3	2	2	1	0	0
		*Drusilla canaliculata* (Fabricius, 1787)	Living	0	0	0	0	0	0	2	4	4	8	8
			Dead	8	8	8	8	8	8	6	4	4	0	0
		*Oxytelus sculptus* Gravenhorst, 1806	Living	0	0	0	0	0	0	0	0	1	6	8
			Dead	8	8	8	8	8	8	8	8	7	2	0
		*Rugilus rufipes* Germar, 1836	Living	0	0	0	0	2	6	8	7	8	8	8
			Dead	8	8	8	8	6	2	0	1	0	0	0
		*Xantholinus tricolor* (Fabricius, 1787)	Living	0	0	0	0	1	3	3	4	4	5	5
			Dead	5	5	5	5	4	2	2	1	1	0	0
		*Bisnius fimetarius* (Gravenhorst, 1802)	Living	0	0	0	0	0	0	0	2	3	7	8
			Dead	8	8	8	8	8	8	8	6	5	1	0
		*Philonthus coprophilus* Jarrige, 1949	Living	0	0	0	0	0	0	2	4	5	5	5
			Dead	5	5	5	5	5	5	3	1	0	0	0
		*Ph. decorus* (Gravenhorst, 1802)	Living	0	0	0	0	0	0	4	7	8	8	8
			Dead	8	8	8	8	8	8	4	1	0	0	0
		*Ph. laevicollis* (Lacordaire, 1835)	Living	0	0	0	0	0	0	3	6	8	8	8
			Dead	8	8	8	8	8	8	5	2	0	0	0
		*Ph. nitidus* (Fabricius, 1787)	Living	0	0	0	0	0	0	5	7	8	8	8
			Dead	8	8	8	8	8	8	3	1	0	0	0
		*Platydracus fulvipes* Scopoli, 1763	Living	0	0	0	1	0	1	4	5	5	5	5
			Dead	5	5	5	4	5	4	1	0	0	0	0
		*Platydracus latebricola* (Gravenhorst, 1806)	Living	0	0	0	0	0	0	3	4	4	5	5
			Dead	5	5	5	5	5	5	2	1	1	0	0
Coleoptera	Silphidae	*Silpha carinata* Herbst, 1783	Living	0	0	0	0	2	8	7	8	8	8	8
			Dead	8	8	8	8	6	0	1	0	0	0	0
		*Phosphuga atrata* (Linnaeus, 1758)	Living	0	0	0	0	1	4	4	5	5	5	5
			Dead	5	5	5	5	4	1	1	0	0	0	0
Coleoptera	Histeridae	*Hister fenestus* Erichson, 1834	Living	0	0	0	0	1	2	1	3	4	8	8
			Dead	8	8	8	8	7	6	7	5	4	0	0
Coleoptera	Aphodiidae	*Aphodius foetens* (Fabricius, 1787)	Living	0	0	0	0	0	4	7	8	8	8	8
			Dead	8	8	8	8	8	4	1	0	0	0	0
		*Teuchestes fossor* (Linnaeus, 1758)	Living	0	0	0	0	2	5	6	6	6	6	6
			Dead	6	6	6	6	4	1	0	0	0	0	0
Hymenoptera	Formicidae	*Ponera coarctata* (Latreille, 1802)	Living	0	0	0	0	0	0	5	6	8	8	8
			Dead	8	8	8	8	8	8	3	2	0	0	0
		*Myrmica ruginodis* Nylander, 1846	Living	0	0	0	0	1	2	6	8	8	8	8
			Dead	8	8	8	8	7	6	2	0	0	0	0
		*Lasius flavus* (Fabricius, 1782)	Living	0	0	0	0	0	0	0	0	1	4	8
			Dead	8	8	8	8	8	8	8	8	7	4	0
		*L. fuliginosus* (Latreille, 1798)	Living	0	0	0	0	1	3	6	8	8	8	8
			Dead	8	8	8	8	7	5	2	0	0	0	0
		*L. niger* (Linnaeus, 1758)	Living	0	0	0	0	0	0	0	0	1	5	8
			Dead	8	8	8	8	8	8	8	8	7	3	0

**Table 2:** LC_50_ of pirimiphos-methyl for the studied invertebrates, their trophic specialization, body mass, and length.

Order	Family	Species	LC_50_ (Mean ± Standard Error), Milligram of Pirimiphos-Methyl Per m^2^	Trophic Group	Average Body Mass (Dry Weight), mg	Body Length, mm
Isopoda	Porcellionidae	*Porcellio laevis* Latreille, 1804	18.24 ± 3.08	s	29	10–20
Lithobiomorpha	Lithobiidae	*Lithobius forficatus* (Linnaeus, 1758)	27.70 ± 2.68	z	21	18–30
Julida	Julidae	*Cylindroiulus truncorum* (Silvestri, 1896)	49.22 ± 10.35	s	42	15–20
		*Megaphyllum* sp.	12.95 ± 1.94	s	47	17–22
Dermaptera	Spongiphoridae	*Labia minor* (Linnaeus, 1758)	7.91 ± 1.94	p	3	4–7
Hemiptera	Pyrrhocoridae	*Pyrrhocoris apterus* (Linnaeus, 1758)	62.63 ± 10.34	p	32	9–11
Coleoptera	Carabidae	*Carabus convexus* Fabricius, 1775	8.74 ± 1.43	z	120	15–18
		*C. granulatus* Linnaeus, 1758	7.58 ± 1.46	z	106	17–23
		*C. hortensis* Linnaeus, 1758	25.25 ± 3.76	z	237	23–30
		*Leistus ferrugineus* (Linnaeus, 1758)	10.65 ± 2.12	z	7	6.5–8.0
		*Pterostichus niger* (Schaller, 1783)	30.29 ± 6.15	z	74	15–21
		*P. melanarius* (Illiger, 1798)	9.62 ± 1.97	z	71	12–18
		*P. oblongopunctatus* (Fabricius, 1787)	18.20 ± 2.80	z	13	9–12
		*Limodromus assimilis* (Paykull, 1790)	9.23 ± 2.58	z	15	10–13
		*Ophonus rufibarbis* (Fabricius, 1792)	22.37 ± 4.15	f	11	7.5–10.0
		*Badister sodalis* (Duftschmid, 1812)	19.24 ± 3.69	z	1	3.9–4.8
		*Harpalus latus* (Linnaeus, 1758)	9.56 ± 1.90	f	12	8.0–10.5
		*H. rufipes* (De Geer, 1774)	28.14 ± 5.68	p	47	11–16
		*H. xanthopus winkleri* Schauberger, 1923	10.27 ± 2.16	f	7	6.0–7.5
		*Amara nitida* Sturm, 1825	12.03 ± 2.81	f	6	6.5–7.5
		*Oxypselaphus obscurus* (Herbst, 1784)	14.67 ± 2.81	z	4	5.0–6.0
Coleoptera	Staphylinidae	*Tachinus signatus* Gravenhorst, 1802	9.00 ± 1.44	z	0.8	5–6
		*Drusilla canaliculata* (Fabricius, 1787)	4.63 ± 1.19	z	0.6	4–4.8
		*Oxytelus sculptus* Gravenhorst, 1806	0.756 ± 0.328	z	0.6	3.5–4.0
		*Rugilus rufipes* Germar, 1836	19.01 ± 3.30	z	5	5.5–7.5
		*Xantholinus tricolor* (Fabricius, 1787)	13.23 ± 1.48	z	8	9–12
		*Bisnius fimetarius* (Gravenhorst, 1802)	2.12 ± 0.70	z	6	6.0–7.5
		*Philonthus coprophilus* Jarrige, 1949	8.58 ± 2.44	z	7	6.6–8.5
		*Ph. decorus* (Gravenhorst, 1802)	9.23 ± 2.58	z	12	11–13
		*Ph. laevicollis* (Lacordaire, 1835)	8.14 ± 2.30	z	10	8.5–14.0
		*Ph. nitidus* (Fabricius, 1787)	9.68 ± 2.66	z	15	10.5–16.0
		*Platydracus fulvipes* Scopoli, 1763	14.64 ± 3.32	z	22	13–19
		*P. latebricola* (Gravenhorst, 1806)	8.02 ± 1.92	z	16	10.5-15.5
Coleoptera	Silphidae	*Silpha carinata* Herbst, 1783	21.91 ± 5.00	p	74	12–23
		*Phosphuga atrata* (Linnaeus, 1758)	18.55 ± 3.22	z	42	10–16
Coleoptera	Histeridae	*Hister fenestus* Erichson, 1834	5.32 ± 1.14	z	6	4–6
Coleoptera	Aphodiidae	*Aphodius foetens* (Fabricius, 1787)	15.14 ± 3.18	s	9	6.0–8.5
		*Teuchestes fossor* (Linnaeus, 1758)	22.12 ± 4.21	s	41	8–13
Hymenoptera	Formicidae	*Ponera coarctata* (Latreille, 1802)	8.98 ± 2.42	p	0.3	2.0–4.0
		*Myrmica ruginodis* Nylander, 1846	14.32 ± 2.58	p	0.7	3.0–4.5
		*Lasius flavus* (Fabricius, 1782)	0.481 ± 0.171	p	0.3	2.5–4.0
		*L. fuliginosus* (Latreille, 1798)	14.96 ± 2.53	p	0.5	3.5–5.5
		*L. niger* (Linnaeus, 1758)	0.603 ± 0.234	p	0.4	3.0–4.5

Note. The manufacturer-recommended dose of pirimiphos-methyl in agrocoenoses is 50 mg/m^2^ or 500 g/ha. Trophic groups: f—phytophages, s—saprophages, z—zoophages, p—pantophages.

## Data Availability

All data are either published with the manuscript or are available on request from the lead author.
